# Effects of continual intermittent administration of parathyroid hormone on implant stability in the presence of osteoporosis: an *in vivo* study using resonance frequency analysis in a rabbit model

**DOI:** 10.1590/1678-7757-2016-0561

**Published:** 2017

**Authors:** Yoshifumi Oki, Kazuya Doi, Yusuke Makihara, Reiko Kobatake, Takayasu Kubo, Kazuhiro Tsuga

**Affiliations:** Hiroshima University, Graduate School of Biomedical & Health Sciences, Department of Advanced Prosthodontics, Division of Dental Sciences, Hiroshima, Japan

**Keywords:** Dental implants, Parathyroid hormone, Osseointegration

## Abstract

**Objective:**

This study aimed to evaluate the effects of continual intermittent administration of parathyroid hormone (PTH) on implant stability in the presence of osteoporosis, using rabbit models.

**Material and Methods:**

Fifteen female New Zealand white rabbits underwent ovariectomy and were administered glucocorticoids to induce osteoporosis, following which they were divided into three groups. The first group received intermittent subcutaneous PTH for 4 weeks until implant placement (PTH1), while the second and third groups received PTH (PTH2) and saline (control), respectively, for 4 weeks before and after implant placement. After intermittent administration of PTH or saline, titanium implants were inserted into the left femoral epiphyses of all animals, and the implant stability quotient (ISQ) was measured immediately after placement to assess the primary stability and at 2 and 4 weeks after implant placement to assess osseointegration. At 4 weeks after implant placement, histological and histomorphometric evaluations were conducted and the bone area around the implant socket was measured as a ratio of the total bone area to the total tissue area.

**Results:**

Regarding primary stability, the ISQ values for the PTH1 and PTH2 groups were significantly higher than those for the control group (p<0.05). Concerning osseointegration, the ISQ values at 2 and 4 weeks were significantly higher for the PTH2 group than for the PTH1 and control (p<0.05) groups. Histological assessments showed a thicker and more trabecular bone around the implant sockets in the PTH2 specimens than in the PTH1 and control specimens. The bone area around the implant socket was significantly greater in the PTH2 group than in the PTH1 and control groups (p<0.05).

**Conclusions:**

Our results suggest that continual intermittent PTH administration before and after dental implant placement is effective for the achievement of favorable stability and osseointegration in the presence of osteoporosis.

## Introduction

Successful implant therapy depends on the achievement of favorable implant stability, which can be divided into primary stability and secondary stability or osseointegration^9^. Both primary stability and osseointegration are affected by different factors, including bone quantity and quality, implant design, and surgical protocols^24^. In particular, the most important factor is the condition of the bone at the site of implant placement^19^. Primary stability decreases at sites with a low bone density, which may result in implant failure^25^.

Osteoporosis is a skeletal disease that causes the systematic loss of bone regarding density and quantity. As mentioned above, the condition of the bone at the implant placement site is strongly correlated with the implant failure rate. Patients with osteoporosis who undergo implant treatment show less favorable outcomes compared with patients exhibiting healthy bone^28^. The most common secondary form of osteoporosis is that induced by glucocorticoid treatment administered for several inflammatory and autoimmune disorders^17^. Glucocorticoids affect the bone quality mainly by decreasing bone formation by a decrease in osteoblastogenesis and an increase in osteoblast and osteocyte apoptosis. Therefore, glucocorticoid-induced osteoporosis is an unfavorable factor regarding implant stability. In a previous study, we showed that glucocorticoid-induced osteoporosis decreased the primary stability of implants and the mechanical strength of the femur in a rabbit model^23^. The phenomenon of poorly primary stability was caused by reduction of cortical bone thickness and mechanical strength.

Currently, the intermittent administration of parathyroid hormone (PTH) for enhancing bone formation and improving bone quantity is clinically approved. Some animal studies have reported that intermittent PTH administration is effective in promoting bone remodeling and increasing the trabecular bone mass^10^ ^,^ ^11^. PTH affects cancellous bone remodeling by promoting the formation of osteoblasts and suppressing their apoptosis^2^ ^,^ ^16^. Furthermore, it increases the thickness of not only trabecular bone, but also cortical bone^15^. Therefore, intermittent PTH administration can be effective in improving the bone density at the implant placement site and achieving favorable primary stability and osseointegration in patients with severe osteoporosis, including that induced by glucocorticoids. Corsini, et al.^5^ (2008) reported that intermittent PTH administration enhanced secondary stability in normal healthy rabbits. Almagro, et al.^1^ (2013) reported that osseointegration could be improved by intermittent PTH administration in rabbit models with osteoporosis.

In these studies, however, intermittent PTH administration was initiated after implant placement; furthermore, only secondary implant stability or osseointegration was evaluated. Therefore, the effects on primary stability remained unclear, considering the bone quality at the implant placement site was not improved by prior intermittent PTH administration. On the other hand, our previous study assessed the effects of intermittent PTH administration initiated before implant placement in rabbit models with osteoporosis^21^. Thus, the bone condition was improved before implant placement and favorable primary stability was achieved. However, secondary stability was not evaluated. Therefore, few studies have evaluated the effects of PTH therapy on osseointegration after the achievement of favorable primary stability. This study aimed to evaluate the effects of continual intermittent PTH administration before and after dental implant placement on primary stability and secondary stability in the presence of osteoporosis induced in rabbit models by ovariectomy and glucocorticoid administration.

## Material and methods

### Ethics

All animal experiments were conducted in accordance with the current version of the Japan Law on the Protection of Animals. This study was approved by the Research Facilities Committee (A16-3). All surgeries were performed under general anesthesia, and all efforts were made to minimize suffering during the experimental period.

### Animals and experimental design

**Figure 1:**
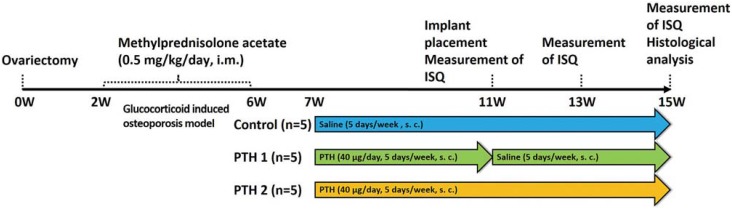
Study design

Fifteen 17-week-old female New Zealand White rabbits (3.0-3.5 kg body weight) were used in this study. The experimental design is shown in Figure 1. All animals initially underwent ovariectomy, and, 2 weeks later, they received intramuscular injections of methylprednisolone acetate (0.5 mg/kg/day) (Depo-Medrol^®^, Pfizer, New York, New York, USA) for 4 consecutive weeks to induce osteoporosis^3^ ^,^ ^4^. Seven weeks after ovariectomy, the animals were divided into three groups. The first group received subcutaneous PTH (40 μg/day, 5 days/week) (Forteo^®^, Eli Lilly, Indianapolis, Indiana, USA) for 4 weeks (PTH1 group) until implant placement, then saline was administrated for 4 weeks. The second group received subcutaneous PTH for 4 weeks before and after implant placement (PTH2 group), and the third group received saline vehicle solution for 4 weeks before and after implant placement as osteoporosis (control group). The study end point was at 4 weeks after implant placement.

### Implantation procedure

**Figure 2:**
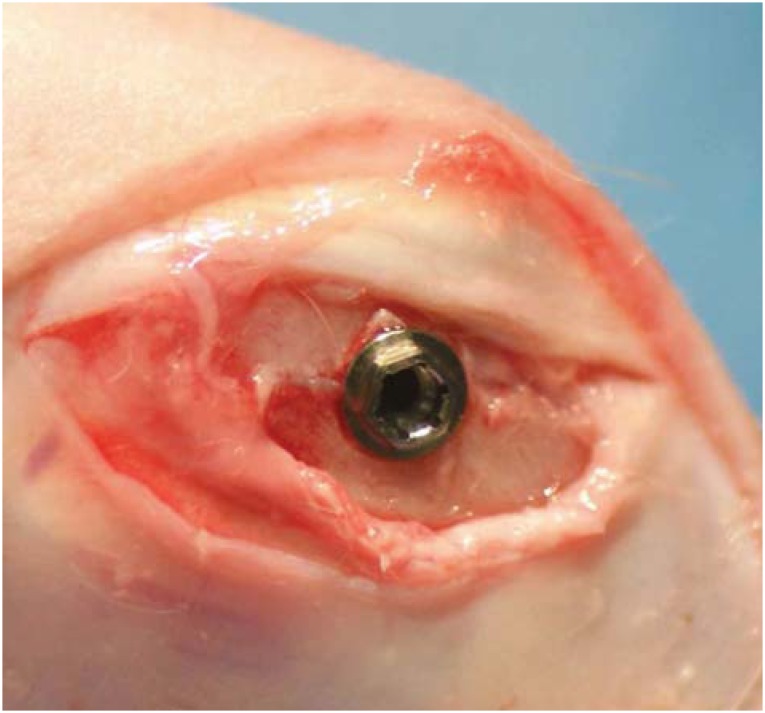
Implant placement in a rabbit model of osteoporosis. The socket is created in the distal epiphysis (knee joint) of the left femur. After the knee joint was exposed, an implant surgical system with a rotary speed not exceeding 800 rpm was used for consecutive applications of a 2.0-mm round drill, 2.0-mm twist drill, 3.0-mm pilot drill, 3.0-mm twist drill, and countersink drill. Following the socket preparation procedures, implants were inserted until the color indicator was level with the bone ridge

All procedures were performed under anesthesia with sodium pentobarbital (10 mg/kg, i.v.; Somnopentyl^®^, Kyoritsu Seiyaku Corporation, Chiyoda-ku, Tokyo, Japan). Implant sockets were prepared in the distal epiphysis (knee joint) of the left femur according to the GC protocol in the manufacturer's instructions. Briefly, after the knee joint was exposed, an implant surgical system (iChiropro, Bien-air, Bienne, Bern, Switzerland) with a rotary speed not exceeding 800 rpm was used for consecutive applications of a 2.0-mm round drill, 2.0-mm twist drill, 3.0-mm pilot drill, 3.0-mm twist drill, and countersink drill. Following the socket preparation procedures, implants (3.8 mm in diameter, 6.5mm in length; SETiO®, GC, Itabashi-ku, Tokyo, Japan) were inserted until the color indicator was level with the bone ridge (Figure 2).

### Measurement of the implant stability quotient (ISQ)

**Figure 3:**
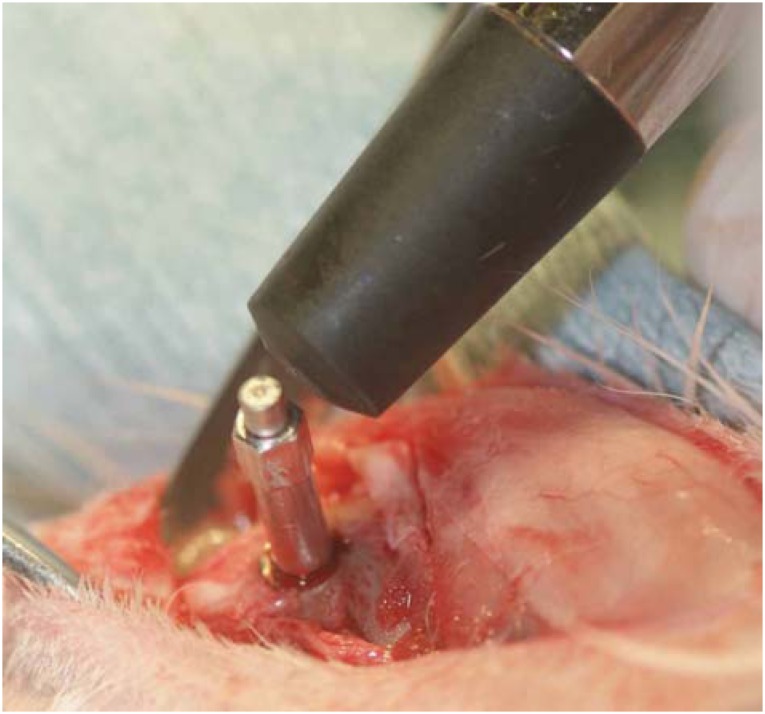
Implant stability quotient (ISQ) measurements. Measurements are performed three times along the short and long axes to obtain mean values for the placed implant

Resonance frequency analysis (RFA) was performed using an Osstell^®^ device (Osstell AB, Gothenburg, Västra Götalands län, Sweden) to measure the implant stability quotient (ISQ) immediately and 2 and 4 weeks after implant placement for the evaluation of primary stability and secondary stability, respectively (Figure 3).

Measurements were performed three times from two different directions, and the values obtained for each implant were averaged. All measurements were obtained using procedures described in a previous study^6^ ^,^ ^7^.

### Histological analysis

Four weeks after implant placement, the animals were sacrificed, the placed implants were removed, and tissue blocks were collected. The tissue blocks* were trimmed and cut using a diamond saw system (400CS, EXAKT Apparatebau, Norderstedt, Land Schleswig-Holstein, Germany) at the center of the implant socket. The Observation section was set at the cross-section. Then, tissue blocks were fixed in 10% neutral formalin for 2 weeks and decalcified with hydrochloride solution (KC-X^®^, FALMA, Shibuya-ku, Tokyo, Japan) for 5 days, dehydrated by a graded ethanol series, cleared with xylene, and embedded in paraffin. Sections with a 5-μm thickness were obtained from each block and stained with hematoxylin and eosin. Histological analysis was performed using light microscopy (BZ-9000, Keyence, Osaka, Osaka, Japan). Histological images were digitized and histomorphometrically analyzed using NIH ImageJ software (National Institutes of Health, Bethesda, Maryland, USA), and the bone area around the implant socket was measured as a ratio of the total bone area to the total tissue area. The regions of interest for the calculation of this ratio were set in the area around the implant socket, at 1.5 mm from its side and at half the vertical distance from the top of the implant shoulder. These regions were selected in accordance with previous studies^21^.

### Statistical analysis

The data obtained were expressed as means ± standard deviations. The values obtained were statistically analyzed using one-way analysis of variance and Tukey's HSD test for multiple comparisons, with significance level set at 5%.

## Results

### Results of the RFA

**Figure 4:**
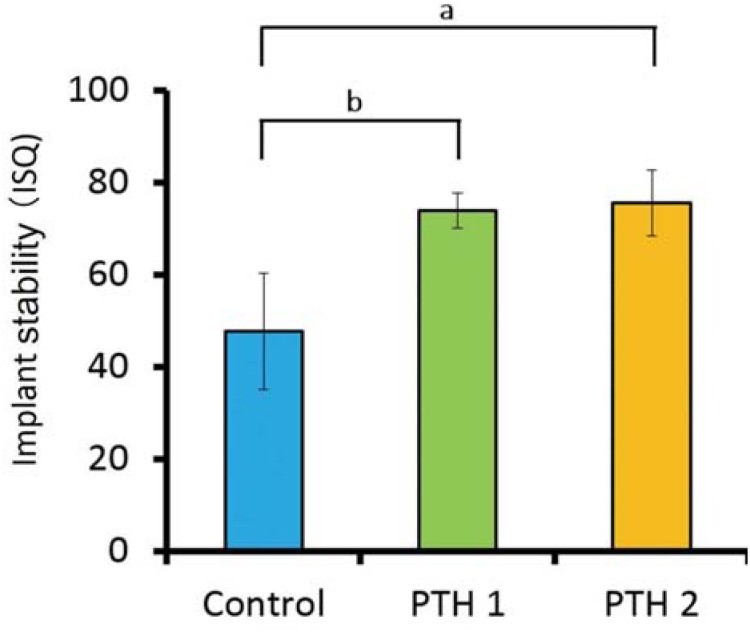
Implant stability quotient (ISQ) values immediately after implant placement; a) p=0.0007. The value for the PTH2 group is significantly higher than the value for the control group; b) p=0.0013. The value for the PTH1 group is significantly higher than the value for the control group

Figure 4 shows the ISQ values obtained immediately after implant placement. The values for the PTH1 (73.9±3.9) and PTH2 groups (75.6±7.1) were significantly higher than the value for the control group (47.7±12.7; p<0.05).

**Figure 5:**
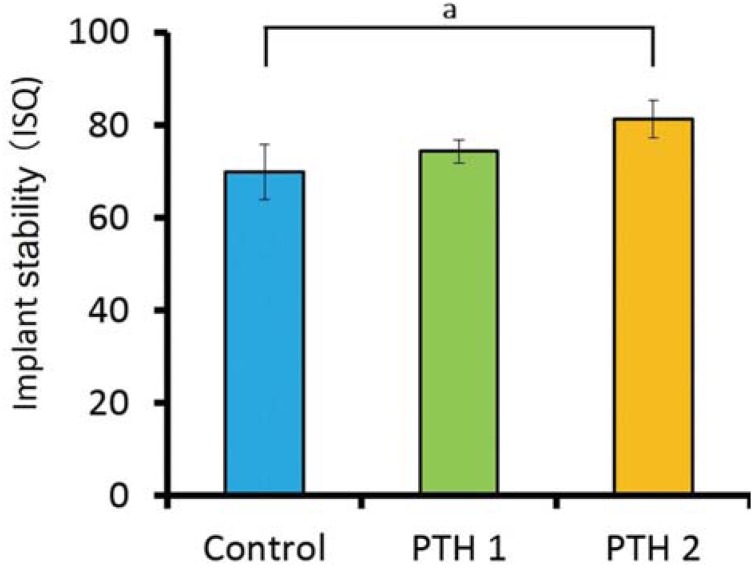
Implant stability quotient (ISQ) values 2 weeks after implant placement; a) p=0.0044. The value for the PTH2 group is significantly higher than the value for the control group

Figure 5 shows the ISQ values obtained 2 weeks after implant placement. The values for the control, PTH1, and PTH2 groups were 70.0±6.0, 74.4±2.5, and 81.4±4.0, respectively, with a significant difference between the control and PTH2 groups (p<0.05).

**Figure 6:**
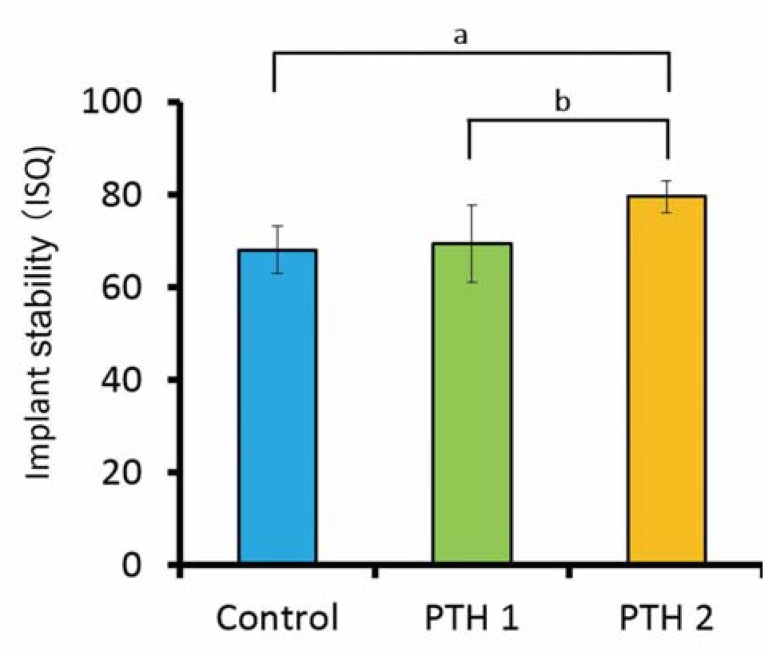
Implant stability quotient (ISQ) values 4 weeks after implant placement; a) p=0.0267, b) p=0.0490. The value for the PTH2 group is significantly higher than the value for the PTH1 and control group

Figure 6 shows the ISQ values obtained 4 weeks after implant placement. At this point, the value for the PTH2 group (79.6±3.5) was significantly higher than the values for both the control (68.1±5.1) and PTH1 (69.4±8.3) groups (p<0.05).

### Histological observations and histomorphometric analyses

**Figure 7:**
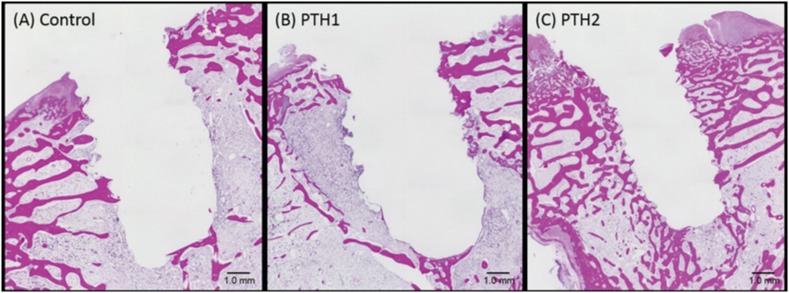
Histological analyses of specimens from the (A) Control as osteoporosis. (B) PTH1: PTH administration for 4 weeks before implant placement. (C) PTH2: PTH administration for 4 weeks before and after implant placement. In the PTH1 and control specimens, trabecular bone is limited to the upper portion around the implant socket; the lower portion is primarily occupied by marrow or fibrous tissue. In the PTH2 specimen, trabecular bone is detected in the upper and lower portions (near the marrow) around the implant socket. (hematoxylin and eosin staining)

Figure 7 shows the findings of histological evaluation. In the control and PTH1 specimens, the trabecular bone structure was limited to the upper portion around the implant socket; the lower portion primarily exhibited marrow or fibrous tissue. In the PTH2 specimen, on the other hand, trabecular bone structure was detected in the upper and lower portions around the implant socket (near the marrow).

The bone area around the implant socket was significantly greater in the PTH2 group (41.7±6.2%) than in the PTH1 (30.8±7.7%) and control groups (25.5±3.8%; Table 1).

**Table 1:** Ratio of bone area

	Bone area % (SD)	Tukey's HSD test
Control (n=5)	25.5 (3.8)^a^	p=0.0034
PTH1 (n=5)	30.8 (7.7)^b^	p= 0.0375
PTH2 (n=5)	41.7 (6.2)	

SD: standard deviation

a Statistically significant difference comparing Control and PTH2 groups

b Statistically significant difference comparing PTH1 and PTH2 groups

## Discussion

Low bone density, such as that observed in patients with osteoporosis, results in poor primary implant stability because of decreased mechanical bone strength at the placement sites. Furthermore, osseointegration is barely achieved at such sites because of the suppression of bone remodeling. In this study, we found that continual intermittent PTH administration before and after implant placement can improve both primary stability and secondary stability, as determined by ISQ values.

Glucocorticoids significantly affect bone quality by suppressing bone formation through the inhibition of osteoblastogenesis and promotion of osteoblast and osteocyte apoptosis^2^ ^,^ ^16^. In our study, ISQ measurements were used to evaluate primary stability and secondary stability. Implant stability is considered an important measurement for evaluating the success of an implant therapy^30^. RFA is a noninvasive method for continuously measuring implant stability in clinical cases^8^ ^,^ ^18^, and we used an Osstell^®^ device to perform it. ISQ values are derived on a scale from 1 to 100, and those for successfully stabilized implants are considered to range from 57 to 82^8^. This device measures ISQ using RFA, which measures the emitting frequency by a vibration transducer attached to the abutment or fixture^14^. These values confirm whether an effective amount of bone is surrounding the implant and whether the bone and implant surfaces have integrated or not^12^ ^,^ ^26^.

Primary stability is defined as the mechanical adaptation between the implant surface and the surrounding bone^27^. In this study, ISQ values for primary stability were significantly higher for the PTH1 (4 weeks before implant placement) and PTH2 (4 weeks before and after implant placement) groups than for the control group. The ISQ value for the control group was only 47.7±12.7, which indicated unfavorable primary stability. These findings suggest the promotion of osteoblast differentiation and suppression of osteoclasts by intermittent PTH administration in poor bone conditions, in accordance with the findings of our previous study^21^. In another study, bone density was significantly decreased after ovariectomy and glucocorticoid administration, as assessed by dualenergy X-ray analysis^4^. In addition, our previous study showed that the mechanical bone strength was lower in rabbit models with osteoporosis induced by ovariectomy and glucocorticoid administration than in a healthy rabbit model. Accordingly, we believe that the primary stability in both PTH groups of our study increased because of an improvement in the bone condition at the implant placement site caused by intermittent PTH administration before implant placement. The aspects consider that intermittent PTH administration before implant placement inhibits osteoclast activity and enhances osteoblast activity, hence the trabecular structure increase at the implant placement portion. Thus, primary stability of PTH1 and PTH2 groups was improved.

Secondary stability as osseointegration is defined as the integration between the implant surface and the surrounding bone. The newly formed bone and bone remodeling at the bone-implant interface and in the surrounding area correlate with RFA measurement^18^. Several studies have reported that the bone condition significantly affects RFA measurements, and that these measurements are related to the supported length of the bone stiffness around the implant socket^8^ ^,^ ^12^ ^,^ ^18^ ^,^ ^22^. In our study, the ISQ value 4 weeks after implant placement was significantly higher for the PTH2 group than for the other two groups. Histological assessments showed a thicker and more trabecular bone in the PTH2 specimens than in the PTH1 and control specimens. In the PTH2 group, newly formed bone was detected not only in the upper portion but also in the lower portion near the bone marrow. In addition, the implant socket could be clearly visualized in this group. In the other two groups, the newly formed bone was limited to the upper portion around the implant socket. Histomorphological analyses indicated that the bone area around the implant socket was significantly greater in the PTH2 group than in the PTH1 and control groups. The ISQ is considered to increase in condition to the stiffness of the bone- implant interface^13^ ^,^ ^26^.

Also, Miyamoto, et al.^20^ (2005) reported a significant correlation detected between the ISQ and bone cortical thickness. This result was in accordance with the ISQ values at 4 weeks, which were high for the PTH group because of new bone formation around the placed implants, caused by the effects of PTH administration during the healing period. The aspects consider that intermittent PTH administration after implant placement accelerate bone formation around the placed implant enhances osteoblast activity. Thus, the secondary stability of PTH2 group was increased. We found no significant differences in ISQ values between the PTH1 and PTH2 groups at 2 weeks after implant placement. Castaneda, et al.^3^ (2008) reported that the effects of glucocorticoids persisted for more than 3 months after discontinuation in a rabbit study. Thus, we considered that the behavior of glucocorticoids persists during the term in this study. On the other hand, the half-life of PTH is relatively short. Therefore, we believe that the bone remodeling caused by PTH administration continues for 2 weeks and ceases at 4 weeks. We observed favorable primary stability in the PTH1 group, although secondary stability was similar to that in the control group. These findings indicate that continual intermittent administration of PTH after implant placement is necessary to promote osseointegration. Various reports have documented the appropriate dosage of PTH for achieving such an effect^5^ ^,^ ^29^. In our study, the PTH dose rate was set at approximately 15-20 μg/kg five times a week, which is within the range used for *in vivo* rabbit studies (1560 μg/kg five times a week)^3^ ^,^ ^21^.

In conclusion, the results of our study suggest that continual intermittent PTH administration is effective for achieving favorable primary and secondary stability in the presence of osteoporosis. It is the authors’ intention to conduct further studies comparing normal healthy models to investigate the detailed effects of PTH on implant stability.
